# „Babysitter“-Nerventransfer vom R. thenaris zum R. profundus nervi ulnaris: Eine Option zum Erhalt der intrinsischen Handmuskulatur bei hohen Läsionen des N. ulnaris

**DOI:** 10.1007/s00064-021-00733-8

**Published:** 2021-09-17

**Authors:** Clemens Gstoettner, Stefan Salminger, Gregor Laengle, Bernhard Gesslbauer, Wolfgang J. Weninger, Lena Hirtler, Oskar C. Aszmann

**Affiliations:** 1 Klinisches Labor für bionische Extremitätenrekonstruktion, Universitätsklinik für Plastische, Rekonstruktive und Ästhetische Chirurgie, Medizinische Universität Wien, Wien, Österreich; 2 Universitätsklinik für Plastische, Rekonstruktive und Ästhetische Chirurgie, Medizinische Universität Wien, Wien, Österreich; 3 Abteilung für Anatomie, Zentrum für Anatomie und Zellbiologie, Medizinische Universität Wien, Wien, Österreich

**Keywords:** Periphere Nervenchirurgie, Selektiver Nerventransfer, Babysitter, N. ulnaris, N. medianus, Peripheral nerve surgery, Selective nerve transfer, Babysitter, Ulnar nerve, Median nerve

## Abstract

**Operationsziel:**

Ziel dieser Operation ist eine frühzeitige Innervation der intrinsischen Handmuskulatur durch Fasern des N. medianus, um einer irreversiblen Atrophie des Muskelgewebes vorzubeugen. Der Nerventransfer erfolgt mittels Babysitter-Interponat, welches jeweils End-zu-Seit an Spender- und Empfängernerv koaptiert wird. Der Eingriff wird kombiniert mit einer proximalen Rekonstruktion des N. ulnaris.

**Indikationen:**

Hochgradige Läsionen des N. ulnaris ohne spontane Regeneration, insbesondere bei proximaler Läsionshöhe und/oder später Patientenvorstellung.

**Kontraindikationen:**

Irreversible Denervation der intrinsischen Muskulatur; Schwäche oder Ausfall des R. thenaris.

**Operationstechnik:**

Der Zugang erfolgt über dem beugeseitigen Handgelenk durch eine longitudinale Inzision. Der R. profundus des N. ulnaris sowie der R. thenaris des N. medianus werden nach Spalten des Retinaculum flexorum dargestellt. Es erfolgt eine Verbindung der beiden Nerven über ein autologes Interponat, welches jeweils in End-zu-Seit-Manier über ein epineurales Fenster an den Spender- (R. thenaris) und den Empfängernerv (R. profundus) koaptiert wird. Dies ermöglicht die zeitgerechte Regeneration einiger motorischer Medianusaxone in die intrinsische Muskulatur, um einer irreversiblen Degeneration vorzubeugen. Aufgrund der End-zu-Seit-Nervennaht wird der Schaden des Spendernervs auf ein Minimum reduziert. Durch die gleichzeitig durchgeführte Rekonstruktion des N. ulnaris auf Höhe der Läsion wird im späteren Verlauf auch eine autochthone Reinnervation der intrinsischen Muskulatur ermöglicht.

**Weiterbehandlung:**

Postoperativ werden Laschen eingebracht und ein steriler Handverband angelegt. Erster Verbandswechsel und Zug der Laschen am ersten postoperativen Tag, Nahtentfernung in der Regel nach 2 Wochen. Bereits nach 1 Woche kann die ergotherapeutische Beübung zum Erhalt der Gelenkbeweglichkeit erfolgen. Nach den ersten Zeichen der motorischen und/oder sensiblen Reinnervation erfolgt eine zielgerichtete Physiotherapie zum Wiedererlernen der alltäglichen Handfunktion.

**Ergebnisse:**

Diese Technik wurde bisher an 3 Patienten mit hochgradiger Läsion des N. ulnaris vorgestellt. Bei einer Follow-up-Zeit von 6 Jahren konnten alle Patienten Muskelkraft von ≥ M3 erlangen, mit allgemein gutem bis exzellentem Ergebnis anhand der modifizierten Bishop Rating Scale.

## Abstract (en)

**Objective:**

The objective of this surgery is to achieve early reinnervation of the intrinsic hand muscles through axons of the median nerve, preventing irreversible atrophy of the muscle tissue. The nerve transfer is achieved via a babysitter graft, which is sutured end-to-side to the donor as well as the recipient nerve. The procedure is carried out in combination with a proximal reconstruction of the ulnar nerve.

**Indications:**

High-grade lesions of the ulnar nerve without spontaneous regeneration, particularly when lesions are located proximally and/or when patients present late.

**Contraindications:**

Irreversible denervation of the intrinsic muscles; weakness or palsy of the thenar branch.

**Surgical technique:**

The approach is taken through a longitudinal incision over the volar wrist. The deep branch of the ulnar nerve as well as the thenar branch of the median nerve are visualized after transection of the flexor retinaculum. An autologous graft is then placed between the two nerves, sutured to the donor (thenar branch) as well as the recipient nerve (ulnar deep branch) via an epineural window in an end-to-side manner. This facilitates timely regeneration of motor axons from the median nerve into the intrinsic muscles, thereby preventing irreversible degeneration. Through the end-to-side nerve coaptation, damage to the donor nerve is reduced to a minimum. At the same time reconstruction of the ulnar nerve is performed proximally to the lesion, facilitating original reinnervation of the intrinsic muscles at a later time.

**Postoperative management:**

Postoperatively, Penrose drains are placed and a sterile hand dressing is applied. Drain removal and dressing change are performed on the first day, suture removal after 2 weeks. Physical therapy for mobility of the joints can be started as early as 1 week after surgery. After the first signs of motor and/or sensory reinnervation, a targeted retraining of daily skills should be initiated.

**Results:**

This procedure has so far been reported on three patients with high-grade ulnar nerve injury. After a follow-up duration of 6 years, each achieved muscle strength of ≥ M3, with good to excellent overall regeneration according to the modified Bishop rating scale.

## Vorbemerkungen

Hohe Läsionen des N. ulnaris sind definiert als proximal der Abgänge der Muskeläste zu M. flexor carpi ulnaris und M. flexor digitorum profundus [15]. Bei einer Nervenrekonstruktion kann die Regeneration der Axone bis zur intrinsischen Muskulatur der Hand mehrere Monate dauern. Je nach Höhe der Läsion und der vergangenen Zeit seit dem Trauma kann es daher vorkommen, dass eine rechtzeitige Reinnervation der intrinsischen Handmuskulatur nicht mehr möglich ist. Dies ist darin begründet, dass Muskeln bzw. deren motorische Endplatten nach einer Denervationszeit von etwa 12 bis 18 Monaten irreversibel atrophieren und daher nicht mehr funktionell reinnervierbar sind [4, 11].

In den letzten Jahrzehnten wurden für verschiedene Nervenläsionen spezifische distale Nerventransfers beschrieben, welche den Vorteil bieten, die denervierte Muskulatur frühzeitig mit funktionellen Axonen zu versorgen und dabei eine präzise motorische Reinnervation bestimmter Zielmuskeln ermöglichen [18]. Bei hohen Läsionen des N. ulnaris wurde unter anderem ein motorischer Nerventransfer vom N. interosseus anterior an den R. profundus nervi ulnaris in der Literatur beschrieben [2]. Der Nachteil solcher Transfers ist jedoch, dass einerseits unweigerlich ein Spendernerv geopfert werden muss und andererseits eine Reinnervation mittels kognitiv getrennter Axonen erfolgt, was die funktionelle Rehabilitation für den Patienten erschwert.

Falls die proximale Rekonstruktion des geschädigten Nervs möglich ist, bietet ein „Babysitter“-Nerventransfer eine sinnvolle Alternative. Dieses Konzept wurde erstmals von Terzis et al. in der Fazialischirurgie vorgestellt [8, 17]. Bei einseitiger Fazialislähmung kann ein partieller Transfer von etwa 40 % des ipsilateralen N. hypoglossus in End-zu-Seit-Manier erfolgen. Einige Fasern des N. hypoglossus reinnervieren somit die Fazialismuskulatur frühzeitig, während später im Verlauf die endgültige Reinnervation durch Fasern des kontralateralen, gesunden N. facialis erfolgt, welche über einen Cross-Face-Nerve-Graft geleitet werden. Während die N.-hypoglossus-Axone das Ziel haben, einer irreversiblen Schädigung der Muskeln vorzubeugen, erfolgt die funktionelle Wiederherstellung der mimischen Muskulatur durch Reinnervation der kontralateralen Fazialisaxone.

In der hier beschriebenen Operationstechnik wird das Babysitter-Prinzip auf Läsionen des N. ulnaris angewandt. Zusätzlich zur Rekonstruktion auf Höhe der Nervenschädigung erfolgt distal ein Babysitter-Transfer vom R. thenaris des N. medianus. Diese Technik wurde erstmals 2016 von Gesslbauer et al. beschrieben. Das Nerveninterponat wird End-zu-Seit sowohl an den R. thenaris als auch an den funktionslosen R. profundus des N. ulnaris koaptiert, was eine Regeneration von Axonen des N. medianus in die intrinsische Handmuskulatur innerhalb von 2 bis 3 Monaten erlaubt, um diese vor irreversibler Schädigung zu schützen. Durch die End-zu-Seit-Technik der Nervenkoaptation erleidet die Thenarmuskulatur keine Funktionseinschränkung durch diesen Eingriff. Im Verlauf erfolgt die endgültige Reinnervation durch die autochthonen Fasern des N. ulnaris, was im Gegensatz zu klassischen Nerventransfers kein kognitives Umlernen nötig macht.

Während die hier vorgestellte Technik eine sinnvolle und risikoarme Variante zur Protektion der intrinsischen Muskulatur bietet, ist zu erwähnen, dass Erfahrungen damit bisher noch limitiert auf wenige Patienten sind [5]. Ein ähnliches Konzept zum Schutz der intrinsischen Muskulatur vor irreversibler Atrophie wurde mittels End-zu-Seit-Transfer vom N. interosseus anterior beschrieben und bereits an großen Patientenkohorten evaluiert [1]. Für diese Technik wurde rezent ein systematisches Review präsentiert mit erfolgreicher Regeneration der intrinsischen Funktion nach Läsion des N. ulnaris bei 92 % der Patienten [3].

## Operationsprinzip und -ziel

Ziel der Babysitter-Technik in der Nervenchirurgie ist die zeitnahe Reinnervation von Muskulatur nach Nervenschaden, um einer irreversiblen Atrophie vorzubeugen. Die Technik wird in Kombination mit einer proximalen Rekonstruktion des geschädigten Nervs angewandt. Der Sinn des Babysitter-Transfers ist es, die Muskulatur am Leben zu erhalten, bis die rekonstruierten Fasern von proximal das Ziel erreichen. In der hier präsentierten Technik erfolgt zusätzlich zur proximalen Rekonstruktion bei N.-ulnaris-Läsionen ein distaler Transfer via Interponat vom R. thenaris des N. medianus zum R. profundus des N. ulnaris. Im Gegensatz zu klassischen Nerventransfers erfolgt die Nervennaht zum geschädigten Nerv End-zu-Seit, um zu gewährleisten, dass die proximalen Fasern des N. ulnaris nach Rekonstruktion noch bis zum Endorgan regenerieren können. Außerdem muss in diesem speziellen Fall auch der Spendernerv (R. thenaris) nicht geopfert werden, da auch hier die Axone über ein epineurales Fenster Seit-zu-End mit dem Interponat verbunden werden.

## Vorteile

- Frühe Reinnervation der intrinsischen Muskulatur verhindert irreversible Muskelatrophie
- In der Regel kein Donor-Defizit durch End-zu-Seit-Nervennaht
- Funktionelle Reinnervation der intrinsischen Muskulatur durch den N. ulnaris, daher kein kognitives Umlernen notwendig

## Nachteile

- Funktion der intrinsischen Muskulatur kehrt erst nach Reinnervation durch den N. ulnaris zurück
- Ein Nerventransplantat ist notwendig (N. suralis, alternativ z. B. N. cutaneus antebrachii medialis)
- Es besteht die Gefahr, den Thenarast des N. medianus zu verletzen
- Ausmaß der Reinnervation über das Babysitter-Interponat kann variabel sein, geringere Sicherheit verglichen zu End-zu-End-Transfers, wie z. B. vom N. interosseus anterior

## Indikationen

- Hochgradige Läsionen des N. ulnaris, bestätigt durch hochauflösenden Ultraschall oder MRT, und ohne spontane Regeneration nach 3 Monaten, insbesondere bei:
- Schädigung proximal im Verlauf des Nervs (Ellenbogen und höher) und/oder
- späte Patientenvorstellung (6 bis 12 Monate nach Schädigung)

## Kontraindikationen

- Irreversible Denervation der intrinsischen Muskulatur (> 18 Monate nach Schädigung)
- Unzureichende Qualität des Spendernervs bei Ausfall oder Schwäche der Thenarmuskulatur

## Patientenaufklärung

- Allgemeine operative Risiken (Nachblutung, Wundheilungsstörung etc.)
- Dauer bis zur funktionellen Reinnervation durch den N. ulnaris je nach Höhe der Rekonstruktion (Nervenwachstum etwa 1 mm pro Tag)
- Mögliche Schädigung des R. thenaris
- Zu erwartendes Defizit durch Entnahme des Nerventransplantats, in der Regel Taubheit im lateralen Fußbereich bei Entnahme des N. suralis
- Notwendigkeit sekundärer Ersatzplastiken bei ausbleibender Regeneration

## Operationsvorbereitungen

- Klinische Untersuchung sowie entsprechende apparative Diagnostik (Ultraschall, MRT, neurophysiologische Untersuchung) zur Feststellung des Ausmaßes der Nervenläsion sowie deren Höhe
- Klinische Untersuchung zur Feststellung der uneingeschränkten Thenarfunktion

## Instrumentarium

- Lupenbrille oder Mikroskop
- Handchirurgisches Set
- Mikrobesteck
- Vessel-Loops
- Monofiles, nichtresorbierbares Nahtmaterial, Größe 9‑0
- Fibrinkleber
- Hautnähte
- Ggf. Nervenstripper zur Suralisentnahme

## Anästhesie und Lagerung

- In der Regel Intubationsnarkose, da auch Nervenentnahme am Bein notwendig sein kann
- Rückenlage mit ausgelagertem Arm
- Betroffenen Arm bis proximal der Läsion abdecken
- Bein für Nervenentnahme von Knöchel bis knapp proximal des Knies abdecken
- Sterile Blutsperre am Arm ist von Vorteil, vorausgesetzt die Höhe der Nervenläsion lässt dies zu

## Operationstechnik

Abb. 1, 2, 3, 4, 5.

**Figure 1:**
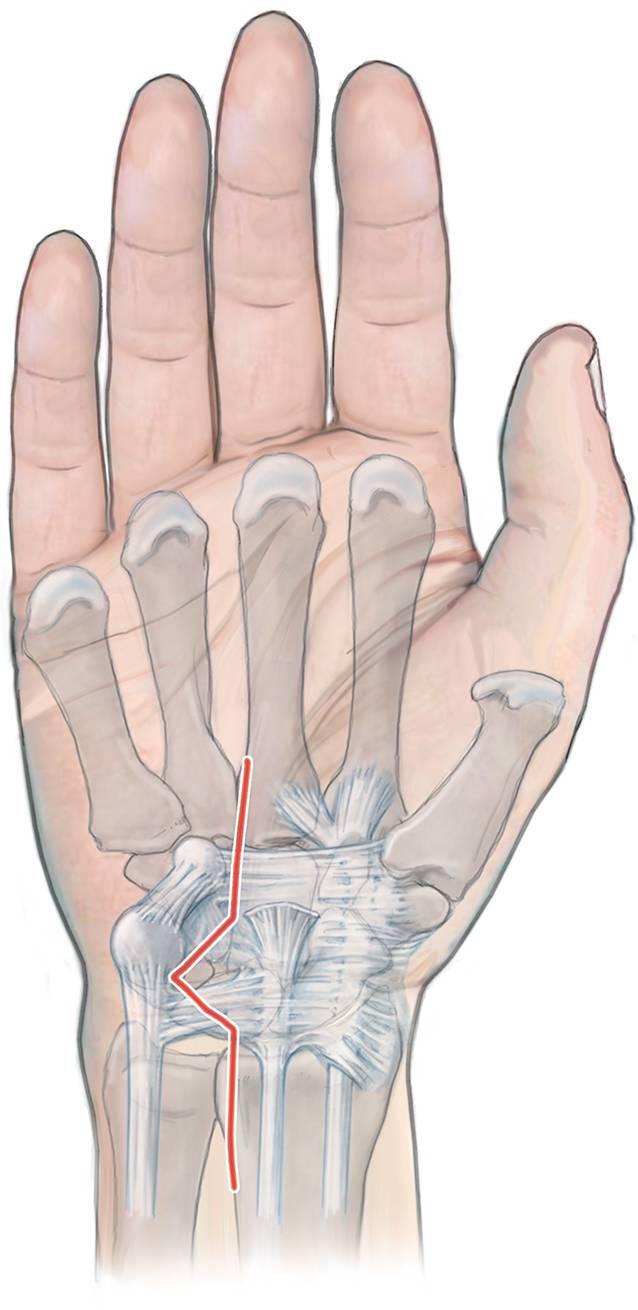

**Figure 2:**
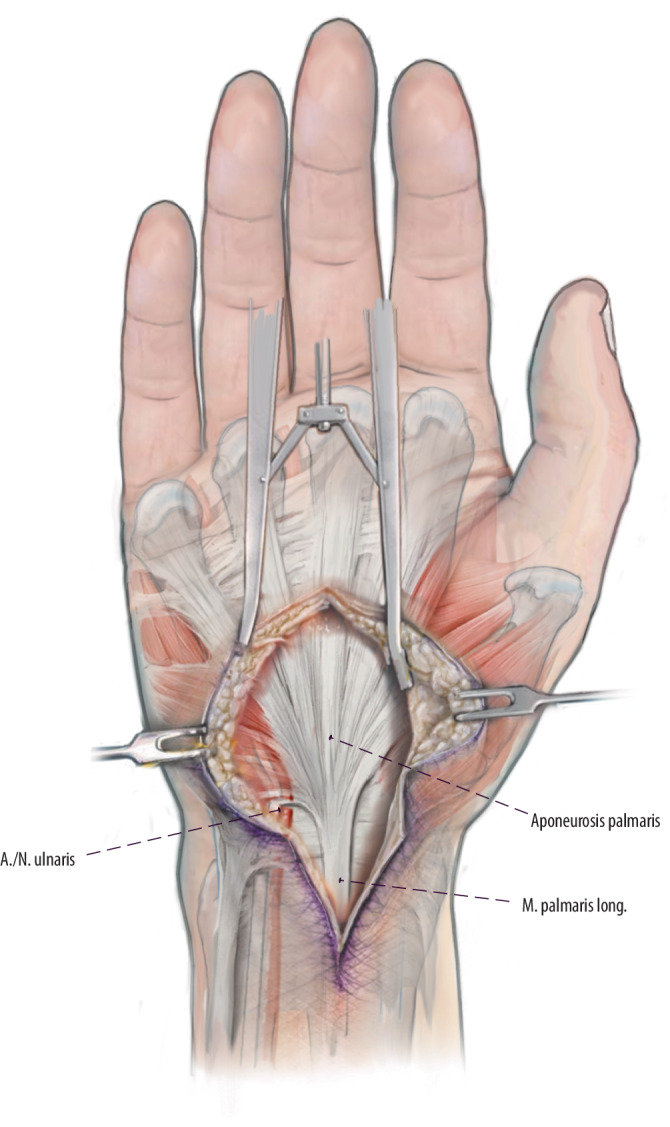

**Figure 3:**
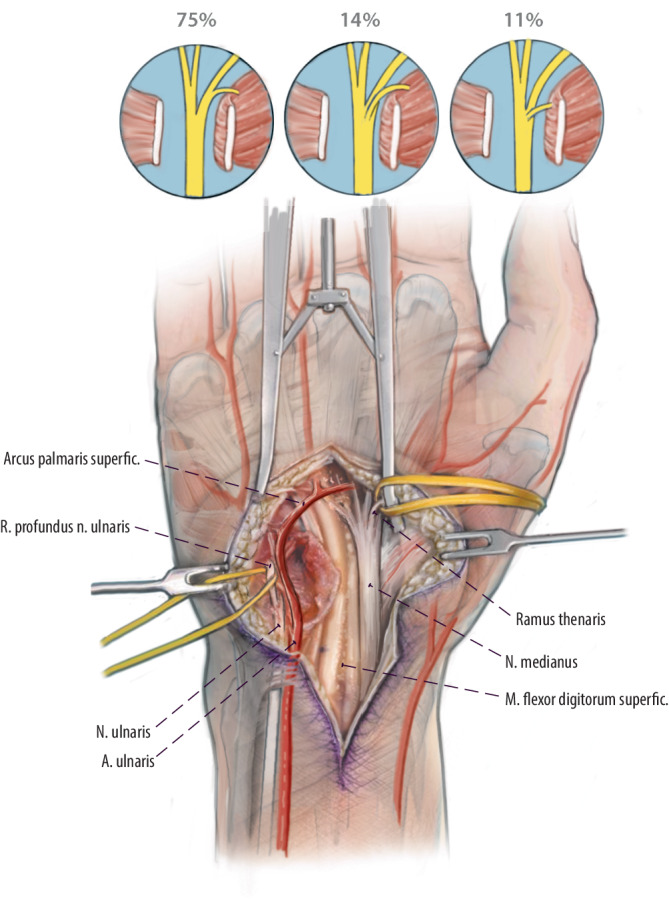

**Figure 4:**
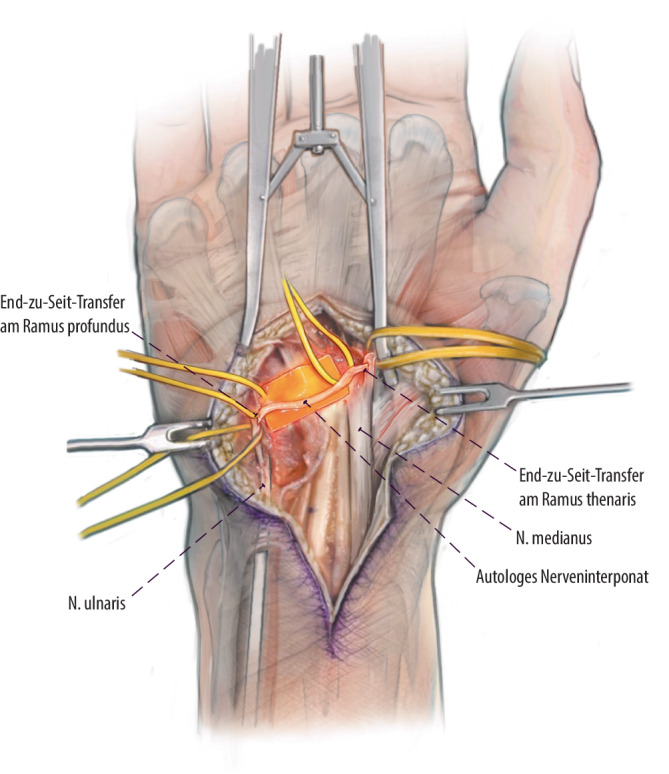

**Figure 5:**
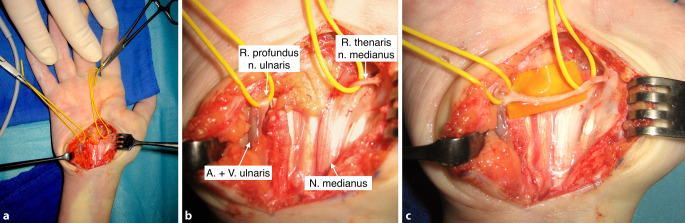

## Postoperative Behandlung

- Drainagelaschen, steriler Handverband, kein Gips notwendig
- Verbandwechsel mit Laschenzug am ersten postoperativen Tag
- Nahtentfernung nach 2 Wochen
- Ergotherapie zum Erhalt der Gelenkbeweglichkeit nach 1 bis 2 Wochen
- Gezieltes sensibles und motorisches Training ab den ersten Anzeichen der Reinnervation durch den N. ulnaris (abhängig von Höhe der proximalen Rekonstruktion)

## Fehler, Gefahren, Komplikationen und ihre Behandlung

- Verletzung des R. thenaris im Rahmen der Operation: Je nach Ausmaß der Schädigung, kann dies zu einem Funktionsverlust der Daumenballenmuskulatur führen. Wird eine scharfe iatrogene Verletzung bereits im Rahmen der Operation bemerkt, muss noch während des Eingriffs die mikrochirurgische Rekonstruktion des Nervs erfolgen. Sollte eine Schwäche nach der Operation auffallen, kann man in der Regel eine spontane Regeneration abwarten (maximal 3 Monate), bevor man den Nerv revidiert.
- Verletzung der A. ulnaris im Rahmen der Operation: Sollte die Arterie iatrogen geschädigt werden, ist eine Rekonstruktion mittels Gefäßnähten anzustreben.
- Ausbleibende Regeneration der intrinsischen Handmuskulatur: Sollte diese kombinierte Operation nicht zu einer ausreichenden Wiederherstellung der intrinsischen Muskelkraft führen, stehen noch sekundäre Ersatzplastiken wie etwa die Lasso-Operation nach Zancolli zur Verfügung [13].

## Ergebnisse

Die Ergebnisse dieser Technik wurden bisher von unserer Arbeitsgruppe an 3 Patienten berichtet [5]. Alle Patienten zogen sich Schnittverletzungen des N. ulnaris am Unterarm zu mit klinisch hochgradigem bis vollständigem Ausfall der intrinsischen Muskulatur (MRC [Medical Research Council] Kraftgrad 1 oder 0). Die Rekonstruktion konnte aufgrund verzögerter Vorstellung jeweils erst zwischen 7 und 12 Monaten nach initialer Verletzung erfolgen. Bei allen Patienten wurde der N. ulnaris mittels autologer Interponate vom N. suralis rekonstruiert sowie zusätzlich ein distaler Babysitter-Transfer vom R. thenaris auf den R. profundus durchgeführt. Die Follow-up-Zeit betrug für alle Patienten 6 Jahre. Die Evaluierung der motorischen Regeneration erfolgte mittels MRC-Grading und durch Messung der Pinch- und Griffkraftstärke mittels entsprechender Dynamometer. Die Sensibilität der Ulnarisfinger wurde anhand der von Mackinnon und Dellon modifizierten Highet–Zachary Scale erhoben [12]. Des Weiteren wurden postoperativ neurophysiologische Untersuchungen durchgeführt, um die Aktivität der intrinsischen Muskulatur nach Stimulation des N. ulnaris und N. medianus zu bestimmen sowie um etwaige Einschränkungen der Thenarfunktion zu quantifizieren. Abschließend wurde eine modifizierte Variante der Bishop Rating Scale zur Feststellung der allgemeinen postoperativen Regeneration erhoben [9].

Postoperativ zeigte sich in den neurophysiologischen Untersuchungen initial Aktivität der intrinsischen Muskulatur nach Stimulation des N. medianus als Zeichen des erfolgreichen Babysitter-Transfers [5]. Im Verlauf stieg die Amplitude der Summenaktionspotenziale deutlich an. Beim finalen Follow-up zeigten sich deutliche Potenziale in der intrinsischen Muskulatur nach Stimulation des N. ulnaris als Zeichen für den Erfolg der proximalen Rekonstruktion. Alle Patienten erreichten nach 6 Jahren eine motorische Funktion der Ulnarismuskulatur von M3–4, bei Ausgangswerten von M0–1 vor der Operation, was schlussendlich auf die erfolgreiche proximale Rekonstruktion zurückzuführen ist. Für die Pinch- und Griffkraft zeigten sich abschließend Werte zwischen 63 und 84 % verglichen zur gesunden Seite. Anhand der modifizierten Bishop Rating Scale konnte die globale Handfunktion für einen der Patienten als gut sowie für die beiden anderen als exzellent beurteilt werden. Es kam bei keinem der Patienten zu einer Einschränkung der Thenarfunktion, und auch sonst traten keine Komplikationen als Folge der Operation auf.
